# A Blockchain-Based Secret-Data Sharing Framework for Personal Health Records in Emergency Condition

**DOI:** 10.3390/healthcare9020206

**Published:** 2021-02-14

**Authors:** Ahmed Raza Rajput, Qianmu Li, Milad Taleby Ahvanooey

**Affiliations:** 1 School of Computer Science and Engineering, Nanjing University of Science and Technology, Nanjing 210094, China; 2 School of Cyber Science and Engineering, Nanjing University of Science and Technology, Nanjing 210094, China; 3 School of Information Management, Nanjing University, Nanjing 210023, China; M.Taleby@nju.edu.cn

**Keywords:** personal health record, emergency access, access control, blockchain, hyperledger fabric, hyperledger composer, auditability, privacy & security

## Abstract

Blockchain technology is the most trusted all-in-one cryptosystem that provides a framework for securing transactions over networks due to its irreversibility and immutability characteristics. Blockchain network, as a decentralized infrastructure, has drawn the attention of various startups, administrators, and developers. This system preserves transactions from tampering and provides a tracking tool for tracing past network operations. A personal health record (PHR) system permits patients to control and share data concerning their health conditions by particular peoples. In the case of an emergency, the patient is unable to approve the emergency staff access to the PHR. Furthermore, a history record management system of the patient’s PHR is required, which exhibits hugely private personal data (e.g., modification date, name of user, last health condition, etc.). In this paper, we suggest a healthcare management framework that employs blockchain technology to provide a tamper protection application by considering safe policies. These policies involve identifying extensible access control, auditing, and tamper resistance in an emergency scenario. Our experiments demonstrated that the proposed framework affords superior performance compared to the state-of-the-art healthcare systems concerning accessibility, privacy, emergency access control, and data auditing.

## 1. Introduction

The Healthcare management system has traditionally been involved with information exchange between patients, business entities such as different hospital systems, pharmaceutical companies, etc. Nevertheless, there has been recent attention towards patient-driven personal health record (PHR), in which health information exchange is patient-mediated. In general, the PHR interoperability involves new requirements and challenges concerning technology, incentives, security and privacy, and governance which should be solved for data sharing issues. Technically, the use of blockchain technology in healthcare management system can provide five mechanisms including: (i) patient identity, (ii) data aggregation, (iii) data liquidity, (iv) digital access rules, and (v) data immutability, which might address such challenges [1,2,3]. However, several management systems exist for healthcare, which controls PHR, incredibly delicate data such as PHR entities [1,2,3]. An ever-increasing selection of medical data estimates actions such as creation, creating, exchanging, and modifying information objects, creating difficulties in tracing malicious activities and security breaches. A PHR is a mechanism for digitally storing a patient’s health data. It needs to allow appropriate access control for manage, track, and restrict their health data [4]. The PHR contains comprehensive health information related to a particular patient like visit dates, prescription drug plans, allergy reports, immunization records, lab results, and so on [5]. Healthcare data sharing is crucial to perform an adequate cooperative manner and care options for patients. In an emergency, the staff requires some essential elementary and relevant health data concerning the patient to enhance the possibility of saving his/her life in sympathetic situations [6]. Some distinct access control policies become limited because no specific policy would admit an emergency staff to obtain the patients’ records. Misuse of the PHR accessing in the emergency is one of the remaining issues in security and privacy [7,8]. In the traditional PHR emergency circumstances, the state-of-the-art frameworks did not confirm the entity’s credentials, unless a single person or group posted a request for the PHR. During the conventional emergency access of the PHR practice, while the Emergency Team (EMT) do actions on the medical records, the malicious users can capture the patient’s health information [9,10]. Most importantly, in the traditional system, it is needed an auditing trail or activity tracking system where the patient can assign some permissions for accessing the PHR. Because when the patient is in an emergency, he/she cannot engage in the access permission approval [11,12]. In the following, we briefly summarized the research objectives of our study.

- Where a traditional emergency system is used to manage the PHRs, it lacks a sufficient control policy tool to limit the access permissions of any third-party person (e.g., doctor/intruder). Therefore, we address this problem by considering security policies using smart contracts which can limit the access permissions to PHRs in an emergency condition.
- Since there is a lack of tracking PHRs in traditional emergency systems, we utilized the audit trails in blockchain technology to provide a tracking option that patients can monitor the history of activities to their records.
- In the traditional emergency system, the PHR access permission should be inquired from one or a number of trustworthy individuals (e.g., family members/friends), where an emergency condition occurs, i.e., it takes much time for contacting such persons. Hence, we solve this issue by defining security policies that a patient can assign which type of users (e.g., family doctor) can access the PHR without requiring any inquiry from other persons.

To address such obstacles and ensure the reliability of PHR, we propose a novel management system based on a blockchain network [13,14] that leverages the shared and changeless distributed ledger. Blockchain is a technology to achieve a valid, challenging to tamper ledger over shared servers. Because of the blockchain network-based system’s capability, when the transaction is endorsed, then the transaction is arduous to alter validly. It utilizes several consensus algorithms to reach approval on the new event for the blockchain. In general, blockchain considers the security as mentioned earlier policies to ensure the reliability of generated records, containing events, termed as blocks. Besides, it empowers authoritative participant’s entry and access control and needs to support accountability. Auditing is the significant property of the blockchain. When the transaction is performed, the current block records the transaction with a timestamp, and the participant of the system trails the previous event actions. It records a history of all transactions. This strategy is beneficial for individual persons or medical organizations that require to obtain tamper-proof account records.

Our system uses the Hyperledger composer [15] based blockchain, which could provide an efficient tool for solving malicious access to the PHR, i.e., This is an extensible and scalable data storage in the off-chain and a person-centered mobile and web edge. In this framework, the blockchain is employed to maintain non-repudiation, accountability, and tamper-proof attributes [16]. The delegate re-encryption method is applied to recommend an access control tool that can help granular access authority. The proposed system utilizes the smart contracts [17,18], which allows the owner of the PHR to assign the rules for an EMT or staff member (certified physician) who can obtain permission to access the current information from the PHR by considering the time restriction. In the normal condition, the patient and their family physician can undoubtedly enter the system through a web browser and mobile interface in an application-based hyperledger composer.

The rest of the article is arranged as follows. Section 2 briefly describes the blockchain Network, Hyperledger Fabric, and Composer. Section 3 explains related works. Section 4 introduces the architecture of our proposed framework. In Section 5, we experiment with the proposed framework by implementing it using the JavaScript Object Notation (JSON) in the Eclipse platform. In Section 6, we discuss our experiments by considering various types of attacks and exhibiting the performance analysis. Finally, Section 7 concludes the remarks of our contributions.

## 2. Blockchain Network

Blockchain is a decentralized distributed technology (DDT) [16]. In blockchain, a collection of records that close share or transfer of value and digital assets such as transactions, goods, and services, is designed and managed by a distributed system of computing nodes in the peer-to-peer network. Blockchain is originated from the bitcoin, a technology that is a distributed database and with the continuously growing records regarded as a block, and these records cannot be changed or altered [19]. The main idea of blockchain is to stabilize the integrity, traceability, and accountability of shared data. Distributed Ledger constrains methods including preservation and authentication, which are executed in a network of interacting nodes. These nodes implement and audit software that harmonizes the shared Ledger images between a peer-to-peer network of shareholders, presenting all accountable activities via digital fingerprints or hash codes. Ledger is classified as pervasive and determined in data recording. In the blockchain, each node member has its shared ledger. It generates a transparent, immutable record [20]. A blockchain logs present accuracy for communication acceptance over the health IT environment and audit logs for following inquiries into such permissions and access models’ performance. Based on this functionality, the framework works as a consistent description of authorization to access the electronic health information (EHI). Over the last decade, the researchers have introduced several healthcare management systems based on blockchain for assuring various security purposes [21,22]. Blockchain guarantees that data was not tampered with by malicious attacks and verified multiple data provenance aspects [23]. This technology involves cryptographic techniques, and the blockchain network’s distributed environment ensures all information distribution, which affords the visible, trustworthy digital fingerprint and auditable paths [24].

There are two primary kinds of blockchain, Permissionless and Permissioned Blockchain. A public blockchain is also called Permissionless Blockchain. The first invention of the permissionless blockchain is Bitcoin. A permissionless blockchain is easily accessible and open for reading and writing actions by all participants on the system [25]. It implies that everybody can participate in the system with pseudonymous identification. The user could also read the information or broadcast them and is identified as a part of the consensus mechanism [26,27]. Ethereum also applies a permissionless Blockchain, and anyone can evolve and combine smart contracts over the network, with no limitation forced by the developers. A permissioned blockchain is also called private blockchain. An individual organization performs a permissioned blockchain [28]. Unlike permissionless blockchain, the permissioned blockchain is designed where participants in the network are predefined for read/write actions and forever identify within the system. So, the main difference between permissionless and permissioned blockchain is how a user can have access to the network. In the permissioned blockchain network, implement Byzantine Fault Tolerance (BFT) [29]. The Hyperledger Fabric is sketched for providing the safety of shared ledger technology and empower permissioned.

### 2.1. Hyperledger Fabric

The Hyperledger Fabric is a type of permissioned blockchain technology that works based on an open-source blockchain enterprise entertained by the Linux Foundation [30]. Hyperledger is a constantly prevalent, collective permissioned or private blockchain that attempts at improving blockchain technology through industry applications. Generally, Hyperledger Fabric is a distributed network formulating a peer-to-peer system where every peer has a replicated, consistent copy of the blockchain data structure, particularly a chained index of transaction describing invocation and executions of chain codes. Hyperledger Fabric gives the chance to increase the application range of blockchain technology beyond cryptocurrency trades which distinct various relational database application domains, comprising the management of healthcare information [31].

### 2.2. Hyperledger Composer

The Linux Foundation entertained Hyperledger Fabric projects which the Hyperledger Composer is one of such examples. The business network archive (BNA) is the functional production of Hyperledger Composer, which is inherited from the blockchain Hyperledger Fabric [15].

**Figure 1:**
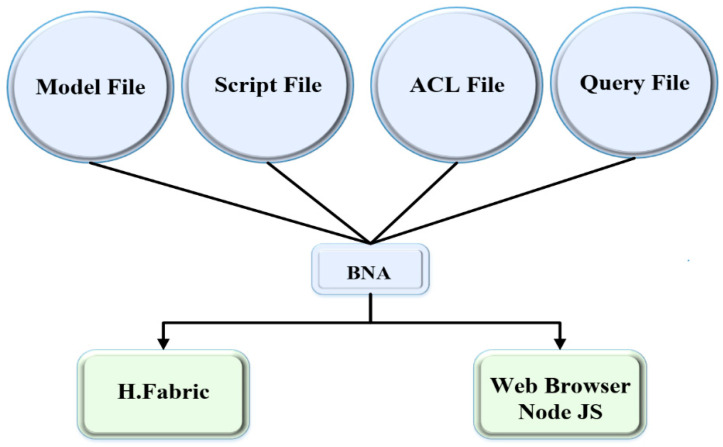
A global architecture for hyperledger composer.

The business network comprises participants, and they are combined through their identifications, as well as, assets that generate on the system; transactions define the exchange of assets. These rules involve executing the transactions called smart contracts, and eventually, all the transactions are saved in the ledger. Figure 1 illustrates the general architecture of Hyperledger Composer. The model file contains three main components: participants, assets, and transactions. The participants are the end-users of the system and can deal with the assets and communicate with other ones by transactions. Assets are usually the variables saved in the network. Transactions are the purposes of the system and are invoked to bring up-to-date the setup. The Script file in the business network determines multiple transaction functions in the system. It is composed of the Java Script (JS) and deals with the business logic, containing which standards of users act and which types of assets are shared. The access control list (ACL) outlines the distinct ranges of participants’ access own in the network. In the ACL file, the participants’ goal is fixed, determining their performance in creating, reading, updating, or deleting the assets. The Query file explains the composition and employment of queries from the system. These remain fixed to extrapolate transactions of the historian, which all of the previous transactions’ records in the network. The Historian record is a registry list fed by the historian record that includes the history of transactions and events performed on the system. While the transaction is processed, the historian record is updated, saving a history of all transactions within a business network. The participants with their identities are involved in submitting the transactions, and historian record assets can be retrieved utilizing composer queries to require particular records.

## 3. Related Works

In this section, we summarize the state-of-the-art healthcare management systems by considering their merits and limitations. Table 1 also shows the merits and limitations of the existing methods.

Guy Zyskind et al. [32] presented the Enigma privacy platform based on blockchain to manage access control and auditing log, privacy, and security objectives, such as a tampered proof record of transactions. Enigma utilizes a multi-party computational model and guarantees data privacy by employing a verifiable secret sharing mechanism. In this platform, researchers claimed that Enigma eliminates the necessity to provide a trusted third-party platform, enabling personal data control anonymously.

Xia et al. [33] presented a framework using the blockchain for protecting data privacy. In this work, the authors suggested a permissioned blockchain system that permits access to data requests by affording knowledge to the information stored in the cloud repository. They employed the data grantors, which authorize the aggregation and review of information, leading to value derivation. Their experimental analysis demonstrated that the system is lightweight, dynamic, and scalable.

A decentralized risk-control system based on blockchain called healthcare data gateways (HDG) system, presented by Xiao Yue et al. [34]. In this system, the patient can own, manage, and distribute his data securely without involving complicated actions, which presents a different latent approach to develop healthcare systems’ ability while preserving patient data confidentiality. From HDG results, it can be concluded that this system is trustable and auditable due to utilizing a decentralized network of peers accomplished by a public ledger.

Azaria et al. [35] developed a medical record sharing prototype called MedRec, the first and only model proposed utilizing some smart contracts to assign appropriate permissions for confidential data sharing. They considered various metadata domains in a single record that distributes individually and may comprise additional limitations such as termination time for data viewership. MedRec provides record versatility and fine-grained, which facilitates patient data sharing and motivations for health data reviewers to maintain the network. In this work, the researchers employed the ledger to maintain an auditable record of medical interactions for patients, healthcare providers, and researchers.

Ichikawa et al. [36] proposed a tamper-resistant mHealth system based on blockchain technology, which provides auditable computing and trustable policies. In this system, they suggested a mHealth network system for cognitive-behavioral medicine in the somnolence (“sleepiness”) disease by developing a smartphone app. Furthermore, they collected the Electronic Medical Records (EMR) from the patients voluntarily via the app saved in JSON format, which was successfully transferred to a permissioned blockchain network called Hyperledger Fabric. Next, the authors analyzed the tamper resistance of the EMRs generated by artificial flaws. Merging blockchain Hyperledger Fabric with mHealth may present an innovative clarification that empowers approachability and data clarity without engaging a third-party.

Xia et al. [37] proposed a new blockchain-based scheme for the trust-less medical data sharing called MeDShare, which protects data records between big-data servers in a trust-less location. In the MeDShare, they utilize a strategy to perform all the events and transmit them into a permanent system, ensuring trust-less and regular auditing policies. Moreover, the authors employed smart contracts and access control policies to efficiently trace the data sharing behavior and prevent access to violated permissions and rules on data.

A data-sharing scheme based on blockchain has been introduced by Hussein [38] for addressing the problems of access control with the blockchain, such as autonomy properties and immutability. In this study, the authors utilized a Discrete Wavelet Transform (DWT) and a genetic algorithm for optimizing the queuing optimization technique. Therefore, it generates a cryptography key for affording access control and immunity, allowing authenticating users in the speedy action.

Dagher et al. [39] introduced a blockchain-based model for providing dynamic, interoperable, and secure access to medical records while protecting patients’ sensitive information. In this system, researchers employed the Ethereum blockchain by defining smart contracts for affording access control and obfuscation of data and applied the cryptographic methods for extra security.

Chen et al. [40] designed a storage system to maintain blockchain-based personal medical data and cloud storage. They employ blockchain as a storage supply chain in which all operations are verified, immutable, and accountable. This system defined the permissions of three types of transactions and composed the block formation and the medical blockchain’s primary function. Furthermore, they introduced a service framework for sharing medical records, which protects medical data management applications without violating privacy policies.

Zhang et al. [41] proposed a secure and privacy-preserving personal health information sharing protocol for diagnosis improvements in the e-Health system based on Blockchain. Moreover, they described the blockchain consensus mechanism, which is the proof of conformance and devised to build validated blocks. Moreover, researchers employed public-key encryption using the keyword search based on the blockchain. A doctor allows to search and access the expected history of health records to enhance the diagnosis after receiving trapdoors from the patient. Besides, they claimed that this eHealth system achieves security, privacy preservation, and a secure search of medical data.

The above state-of-the-art studies are based on blockchain sharing the health record and access control policies. Still, they do not access PHR in an emergency condition. We used a Hyperledger Composer and Fabric for securing the data privacy and auditing trial in emergency access for PHR.

## 4. System Architecture

**Figure 2:**
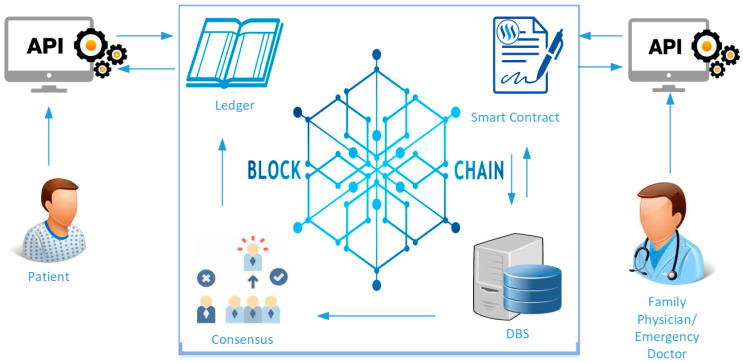
Proposed framework for personal health record (PHR) access control in Emergency.

In this section, we present the proposed emergency access control management system, which utilizes blockchain technology for preserving PHR data privacy. All the data on the blockchain network are shared between the nodes. We develop a system that generates a time-stamped log for all the transactions on the network without engaging a PHR owner or any third party utilizing the Hyperledger Composer-SDK and NodeJS. Moreover, we demonstrate the proposed architecture in Figure 2, which facilitates access control scenario of PHR data by using Hyperledger composer blockchain in an emergency. We first specify the following entities, which involve the process of construction. All the activities are controlled with permissions and the smart contracts that affect data retrieval from the Ledger. In this situation, the patient’s permissions can allow the EMT access to the PHR data. The assumed entities are as follows.

- Patient is a participant who is the owner of the PHR data. A patient defines the access control policies for the PHR data.
- Doctor is a participant, who can log into the system if the patient has granted the permission to him. The PHR owner has to define the policy of access control permission in a smart contract as a family doctor or primary physician.
- Emergency Doctor is a participant who requests emergency access permission while the patient is in an emergency. The proposed framework utilizes an API for granting access according to patients’ rules to the emergency doctor whether he is allowed to access the PHR data or not.
- Rest API, Composer Rest Server creates an Application Programming Interface (API) from the blockchain network that can be efficiently employed by Hypertext Transfer Protocol (HTTP) client for evaluating the permissions.
- Smart Contracts are some transaction protocols that automatically perform, control, and register relevant actions and events according to an agreement’s rules [42]. These are executed on blockchain and administered by a system of peers. They also spontaneously run when specific predefined policies are met. In such a case, the data owner (patient) specifies the access permission in smart contracts.
- Consensus is a mechanism that provides the following core functions in our framework for approving the transaction verifying the patient’s policies. When the transaction is completed, the Consensus accepts the performance and upgrades the main shared ledger to achieve consistent outcomes.
- Ledger is an outcome, tamper confidential records for all the transactions. Transactions are consequences of the smart contracts or requests transmitted from users. Each transaction’s completion is a k-v pair bounded to the state as creates, updates, or delete.

## 5. System Implementation

In this section, we implement the proposed model using Hyperledger Fabric and Hyperledger Composer. During our experiments, we suppose that the user (client) information is retrieved from the JSON, and requested information by utilizing the Rest Client, i.e., Postman server. Every server was formed in the virtual environment Elastic Compute Cloud (EC2) instance on Amazon Web Server (AWS), which operates in the same local personal computer with Ubuntu Linux 18.04.1, single vCPU @ 2.00 GHz, and 32 GB RAM as the details of configuration summarized in Table 2. We employed the Hyperledger composer playground to develop the Business Network Definition. We used Hyperledger Fabric (version 1.2) an open-source project hosted by the Linux foundation. Moreover, we utilized Docker (version 1.12.1), Oracle Virtual Box (version 5.1.22), and Docker compose (version 1.5.2) to set up Docker execution environment. In our framework, ledger’s state is the key-value store database that stores the transaction logs.

Our proposed architecture involves three elements: a patient-centric user interface, a permissioned blockchain, and off-chain storage. Furthermore, we utilized the Hyperledger Composer to build the Business Network Archive (BNA), which defines the network’s properties and abilities. Hyperledger Composer is further used to archive the business network upon the Hyperledger Fabric instance.

This structure includes three main files: Model, Script, and permission (see Figure 1). The model contains three main elements; (i) participants are the actors that can participate in the network (patient, family physician, and emergency doctor), (ii) assets are the data items of the patient’s PHR and some necessary personal information, i.e., they are stored in the variables as regular variables, and (iii) the transactions of participants on the assets through the network. The Script is called “logic.js” which describes several transactions that happened on the system. It maintains the confirmation and validation of the participants, assets, and transactions by considering various system access levels. Moreover, the “permission.acl” contains access control policies in which participants’ rules are defined, i.e., the participant can use the patient’s data in a particular situation (see Table 3). The patient explains the rules for accessing the family physician to PHR information while the patient is in a normal condition. For the emergency condition, the patient also describes the procedure of how an emergency doctor can access using the certified license number. Emergency doctor triggers the smart contracts and receives PHR items with the “emergency access time constraints” function. When the time limit is completed for allowing which emergency doctor could not have access to the system, another essential aspect of Hyperledger Composer is a query file that expresses the formations and policies. Queries are established to generalize activities or actions from the historian, where all the previous records are available through the PHR in the Ledger.

**Figure 3:**
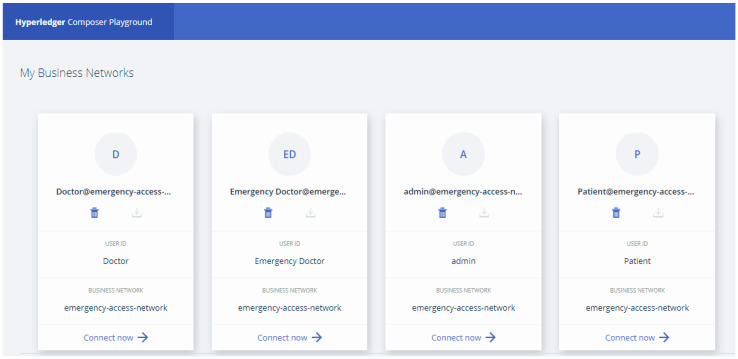
Defined network cards for participants in the hyperledger composer playground.

As depicted in Figure 3, after defining the participant cards in the “My Business Networks” section, we executed the BNA on the Hyperledger Composer. In this case, each network card is utilized to join the system, and identify the kind of participant. These cards regularly have a further organized range of permissions in the network. However, the patient could also complete high-clearance functions (adding or deleting) for participants such as family physician and emergency doctor. This kind of cards determines the node that correlates the identifications to the network and permits to authorize participants.

In our system, new users (family physician and Emergency doctor) with proper identification information can join as a participant at different times. To accomplish an appropriate position, admin manages the participants’ permissions using an alignment of Hyperledger Composer consortium. During the access control management, the characteristics or duties are performed, which kind of transactions specified in “permissions.acl” file. In our proposed framework, the assets are the PHR items such as personal data, test results, and prescribed medicine, etc., which already are stored in the assets registry. In our policies, it is assumed that the transactions are the enrollment processes for the participants and procedures for PHR data item like, “getpatientlabtest” and “getpatienttreatmentdrugs”. Besides, each particular record and its details of the PHR data will be shown on the network. There are four services in this network, including three registration procedures (for the patient, family physician, and emergency doctor), and one function for getting patient’s data from the system. Participants can utilize these functions as a transaction trigger to access relevant data. Each participant’s role depends on the conditions that are predefined by the data owner in the “permission.acl” file.

- Participants Registration: Script file comprises the blockchain transaction processing function (TPF) which is triggered and participant (patient) input parameters consist of the *Card*_*Id*, *First*_*name*, *Last*_*name*, *Address*, *Patient*_*Id*, *Emergency*_*Access*_*Time*_*Costraints*. In the case that the participant is a family doctor, the input parameters include: *Doctor*_*Id*, *First*_*name*, *Last*_*name,* and for the emergency doctor input parameters consist of the *Emerency*_*doctor*_*id*, and *License*_*number* only in an emergency situation and requests access permission by using API to the network. When the participant triggers the function from API, then the node server will explore its endpoint matching scheme in the file “app.js”. After considering all the parameters of the Transaction Processing Function (TPF) which are already saved in the file “network.js”. All TPFs can employ the Hyperledger Composer NodeSDK functions for the registration of patient in the network as a participant. Later, it generates an ID card for the participant and saves it in the ID registry. Query 2 expresses the process of retrieving the PHR records from our system in detail.
- Get Patient Data: This file includes the TPF, which is triggered and obtained from the patient’s PHR data, and the emergency doctor input parameters consisting of *Patient*_*Id* (e.g., current *emergency*_*doctor*_*Id* for an emergency condition). When the participant hits the trigger from the *client*_*side* function named “network.getpatientdata”, which is described in the file “app.js.”, later, our proposed system considers the mandatory fields of the process body for requesting access permission. Then, it will send certain documents to TPFs “networkgetpatientdata,” which is explained in the “network.js” file and is exchanged the data according to prescribed rules. The TPF is again utilized the Hyperledger Composer NodeSDK; first, this verifies whether the participant has permission to access the patient’s information and thus delivers PHR data. The smart contracts restrict the access period according to the patient’s time limitation considered for a particular participant. Additionally, this function generates an occurrence of “EmergencyTimeConstratints” which participant could observe through the playground utilizing the admin ID card. From the well-defined time limitation, the current emergency doctor can view PHR items. Emergecy_Access_End_Time is only two hours more than Emergency_Access_Start_Time. This function also gets the level and rechecks by triggering the get_patient_data, and the emergency doctor will catch the message “Access Denied.” Algorithm 1 shows registering a participant (user) in our system, and Algorithm 2 describes how to get PHR from the system.

**Algorithm 1.** Participant Registration1: **Input**: Emergency Doctor ID, License Number 2: **Output**: Emergency Doctor 3:  Emergency doctor ID ← Emergency doctor 4:  License ← Authorized Doctor License Number 5:  Emergency Doctor ID ← Request for the registration to the system 7: **if** (Authorized Doctor License Number match) **then** 8:  Return Success (Register Emergency Doctor) 9: **else** 10:   Return ‘‘Unauthorized Person’’ 11: **end if**

- Get Patient Data: This file includes the TPF, which is triggered and obtained from the patient’s PHR data, and the emergency doctor input parameters consisting of *Patient*_*Id* (e.g., current *emergency*_*doctor*_*Id* for an emergency condition) when the participant hits the trigger from the *client*_*side* function named “network.getpatientdata”, which is described in the file “app.js.” Later, our proposed system considers the mandatory fields of the process body for requesting access permission. Then, it will send certain documents to TPFs “networkgetpatientdata,” which is explained in the “network.js” file, and data is exchanged according to the prescribed rules. The TPF is again utilized the Hyperledger Composer NodeSDK; first, this verifies whether the participant has permission to access the patient’s information and thus delivers PHR data. The smart contracts restrict the access period according to the patient’s time limitation considered for a particular participant. Additionally, this function generates an occurrence of “EmergencyTimeConstratints,” which participants could observe through the playground utilizing the admin ID card. From the well-defined time limitation, the current emergency doctor can view PHR items. Emergecy_Access_End_Time is only two hours more than Emergency_Access_Start_Time. This function also gets the level and rechecks by triggering the get_patient_data, and the emergency doctor will catch the message “Access Denied.” Algorithm 1 shows registering a participant (user) in our system, and Algorithm 2 describes how to get PHR from the system.

**Algorithm 2.** Get PHR1: **Input:** Emergency Doctor ID, Patient ID 2: **Output:** Display the Patient PHR data items 3: Emergency Doctor ID ← Authorized Emergency Doctor 4: Patient ID ← Discover Registered Patient 5:  Get Patient Data ← Authorized Emergency Doctor request to get patient data 6:  Start time ← get the correct time date 7: **if** (Authorized Emergency Doctor request = true) **then** 8:  Result ← check the Emergency Access Time constraint condition according to the start time 9: **else** 10:   Return “Access Denied” 11: **end if**

**Query 1** Patient Data Retrieval1: { “$class”: “org.hyperledger.composer.system.Add Participant”, 2: “resources”: [ { 3: “$class”: “org.example.basic.EmergencyDoctor”, 4: “emergencyDoctorid”: “ED1”, 5: “licenceNumber”: “A1B2aa444” } ], 6: “targetRegistry”: “resource:org.hyperledger.composer. system.ParticipantRegistry#org.example. basic.EmergencyDoctor”, 7: “transactionId”: “f96ff792-b85f-4c9b-b10d-0d02e0b66e91”, 8: “timestamp”: “2019-10-13T21:53:17.399Z” }

The Historian is a database containing the records that include information about the transactions which occurred on the system. When a transaction is performed, the historian record is updated and timestamp, i.e., a history of transactions in a business network. A Historian record is an asset defined in the Hyperledger Composer network namespace. The Historian registry is updated for all approved transactions. Besides, various operations that the Hyperledger Composer runtime can be classified as transactions.
**Query 2** Adding Asset into the System1: {“$class”: “org.hyperledger.composer.system. AddAsset”, 2: “resources”: [{ 3: “$class”: “org.example.basic.TreatmentDrugs”, 4: “treatmentDrugs”: “Special Treatment”, 5: “drugName”: “Disprine”, 6: “formulae”: “Asprine”, 7: “describption”: “High Headache”, 8: “result”: “Effective”, 9: “emergencyAcces”: true, 10: “owner”: “resource:org.example.basic.Patient#P1”, 11: “doctor”: “resource:org.example.basic.Doctor#D1”}], 12: “targetRegistry”: “resource:org.hyperledger.composer. system.AssetRegistry#org.example.basic.TreatmentDrugs”, “transactionId”: “491c6aa6-8d9c-473f-8cdc-bd2fb2fbda68”, 13: “timestamp”: “2019-10-13T22:09:26.488Z”}

As mentioned earlier, our proposed system utilizes the APIs for querying resources and relationships for registering the historian records. When we call a ‘getAll’ function, it will likely return a massive amount of data from the historian records. Thus, query capacity is essential for obtaining a subset of records based on time limitations. It utilizes the query capacity to select records where the transaction timestamps a particular position. We have conducted our proposed framework by generating some queries as depicted in Query 1 and Query 2. After recovering from the emergency, the patient can check the system’s profile and track all the history records updated on the profile.

## 6. Discussion

In this section, we discuss the proposed framework’s performance concerning auditing, security and privacy, response time, and accessibility.

Does the proposed model provide a secure access control system for PHR data in emergency condition? To answer this question, we applied the Hyperledger Composer based on Hyperledger Fabric, which affords some permissions for participants that allow limited access during an emergency condition. The use of blockchain technology can enhance the security and accessibility of the PHR by different participants in our proposed model while patients are in the emergency concerning confidentiality, non-repudiation, authenticity, and accountability.

Are there any alternatives for malicious attackers to access a patient’s PHR? The answer to this question is, our framework guarantees the patient’s privacy by presenting expediency for designating well-arranged access control to the PHR. Furthermore, it limits the user’s access to the PHR by employing smart contracts. Our mechanism’s access rules essentially concentrate on the purpose, what data object, and which activities they have to perform. In our framework, patient predefined access permissions rules such as read, write, update, delete, and period to share their PHR by smart contracts on the blockchain without the lack of control. Smart contracts can be executed on the blockchain network once all the conditions are met. We proposed that patient can empower access to his/her PHR only under predefined conditions of an appropriate type and for a provided time limit. The smart contract stored directly on the blockchain confirms whether data requestors match these circumstances to access the particularized data. If the requestor does not have access permission, the framework will respond with a message unauthorized user. In the proposed framework, we perform security policies according to the specified participant’s IDs. Hence, it prevents the PHR data from being accessed by malicious users.

Does the proposed system provide auditing during the PHR access in the emergency department? To answer this question, we utilize the historian record, which provides the auditing facility to trace the registered records and history of the PHR data. The Historian record is used only via the patient after his cure from the hospital. It can track and trace all the activities done with his/her PHR in an emergency condition. In other words, various types of actions through the proposed system can be outlined using the historian records.

Our framework ensures the patient’s privacy by affording feasibility for defining granular access control across his/her PHR data. Moreover, it considers access control management by combining smart contracts. In the Hyperledger composer network, the proposed model performs based on the specified participant’s identities. Therefore, there are no ways to access the PHR data for malicious users. Channels in the HF are constructed according to access policies that dictate access to the channel’s stores, such as smart contracts, transactions, and ledger states. Thus, these channels consist of nodes in which the privacy protection and confidentiality of PHR are defined. Our proposed framework protects the PHR data against ransomware and similar security breaches such as unauthorized access. Because it is the decentralized network topology and does not have a single point of failure or central repository for intruders to infiltrate, the emergency doctor has just short, timely access to the system. After the time limit of his/her access data, the emergency doctor could not access the PHR data. Blockchain technology makes the process of adopting the system much simpler and less costly. The implementation facilitates improved security, privacy availability, and auditing by storing access control lists and logs directly on the blockchain. Each attempt to access a record is verified in the access control list and subsequently logged before access is granted to the user. The system introduces a new standard way of managing access control in the emergency condition and auditing across several participants. The experiments confirm that our framework provides better efficiency compared with the traditional emergency access system. Besides, the patients get the historian records for the audit trail and check the access control policies whether their PHR data have not to breach after recovering from the emergency condition. This work presents an implementation of a blockchain framework for improving auditing and privacy measures of PHR systems.

**Figure 4:**
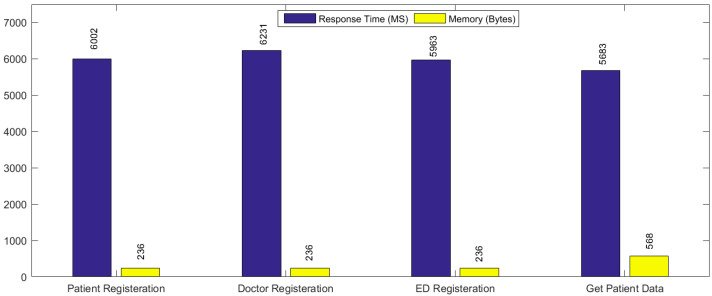
Performance Evaluation of the proposed framework.

What is the difference in the response time efficiency between the proposed framework and the traditional emergency system? The answer to this question is, our proposed system preserves the PHR against data violations while being manipulated by malicious users. Figure 4 depicts the evaluation and performance of our system based on time efficiency and memory. Since we used the smart contracts in our proposed framework, it affords various properties such as time control, verification, and classification that reduce the response time during the processing of queries. In References [11,12], researchers introduced a framework based on trusted members in emergency contact for accessing patient’s information. However, there is no third party or trusted member in the contact list for an emergency condition in our system because we employed the Hyperledger composer while the patients define the access rules/policies in smart contracts for the emergency doctor. Therefore, the emergency doctor can receive requested information in less than a few seconds from our system. In the traditional system [11,12], the average response time for processing text messages and calls to the trusted members is “7188” minutes. Moreover, trusted member affords the reply to the emergency team for allowing the PHR item access, which is (8 min) for receiving calls and messages response-time. Moreover, average registration time of our system is “6900 ms” and getpatientdata average time is “6000 ms”. For responding to the emergency doctor, the average time is “15,000 ms” to “18,000 ms”. These results demonstrate that our proposed framework provides accessibility to the PHR data items without approval by trusted members in the contact list for an emergency. In our experiments, the average response time has been decreased in an emergency condition as compared to the aforementioned traditional system for approving the information of PHR. To provide a comparative analysis, we have evaluated the existing blockchain-based health systems [34,39,40,41,42] considering their strategies for designing security policies. In other words, we conducted a benchmark study to investigate the capabilities of our framework and other systems regarding immutability, identity management, smart contracts, and data auditing. Table 4 depicts the outcome of the benchmark study. We have chosen the parameters that impact the system performance during our analysis. Since we have developed our framework using the Hyperledger composer using the aforementioned policies, it reduces the system’s overall overhead. Note that most of the existing systems work based on EMR functionality (except References [40,41]). Therefore, our framework provides the security policies for PHRs that can improve healthcare system usability in emergency cases.

## 7. Conclusions

In this study, we proposed a new access control framework, which preserves PHR data privacy where a patient is in an emergency condition. Systematically, it works based on the permissioned blockchain Hyperledger Fabric and Hyperledger Composer. In this framework, we utilized the smart contracts in blockchain technology to provide security policies that patients can manage the access rules of other participants in the healthcare system using the consortium strategy. Besides, our system affords the historian records for auditing that stores the history of transactions while patients are in an emergency. Moreover, they can trace the history of the records held by other participants (doctors) after recovery. We experienced our framework using the Hyperledger Composer playground to evaluate its performance of our framework. Our experimental results demonstrated that this framework assures the secret data sharing of the PHR by considering the immutability, auditing, and emergency access control policies.

Our proposed framework not only provides security policies for controlling the access permissions to the PHRs during the emergency condition but also enables the health management system to eliminate the time of emergency contact. However, there exist some limitations which should be addressed in future works. Since our framework is at the prototype stage, we should test it by engaging different groups of participants and take their feedback into account during the maintenance stage. Moreover, because the PHRs are exchanged/shared among different participants (or agencies), a standard like HL7 FHIR is needed to guarantee the security of data sharing implementation.

## Figures and Tables

**Table 1:** Existing blockchain healthcare systems.

Blockchain Systems	Health Data	Merits	Limitations
Xia et al. [33]	Electronic Medical Record	To adequately pursue the execution of the information and revoke access to offending nodes on the exposure of breach of permissions on information.	Participants’ transactions are intended to support various, but limited events for user transaction instances not considered for.
Xiao et al. [34]	Healthcare data	Affords anonymization, productive interaction among HDGs, and data reinforcement and improvement utilizing cloud.	It is inadequate to process information and executes computations without exposing information.
Azaria et al. [35]	Electronic Medical Record	Provides reliable access, perpetual log, and complete services. It also eludes a single point of failure	Does not recognize contract encryption, obfuscation, scalability, and auditability. The scheme demands to be extended for complicated situations concerning healthcare data.
Ichikawa et al. [36]	Electronic Medical Record	Hardy against network faults such as assigned node down.	Vulnerable to attack.
Xia et al. et al. [37]	Medical data	Ensures data provenance, security, and user verification. It provides remote access and data access revocation.	Omitted data revealing concerns.
Hussein et al. [38]	Electronic Medical Record	Enhances overall security and access control, allows fast verification process, and further accountability.	This would support expand system devices and enhance security.
Dagher et al. [39]	Electronic Health Record	Concentrates on protecting patient’s security and privacy utilizing cryptographic techniques and allows access control.	Absorbs computational energy due to a large number of applied smart contracts.
Chen et al. [40]	Personal Medical Data	Patients control their personal medical data.	Interoperability is not examined across various healthcare companies.
Zhang et al. [41]	Personal Health Information	Protected records of PHI are traced employing the consortium blockchain, while the private blockchain reserves the PHI.	The data location might be modified so the old URL cannot be altered, and a novel URL needs to be created.

**Table 2:** Implementation Development Environment.

Component	Description
CPU	Single vCPU @ 2.00 GHz
Operating System	Ubuntu Linux 18.04.1 LTS
Memory	32 GB
Hyperledger Fabric	Version 1.2
Docker	Version 1.12.1
Oracle Virtual Box	Version 5.1.22
Docker-Compose	Version 1.5.2

**Table 3:** The defined access control policies in the “permission.acl” file.

Permission Rules for Limiting Access to the PHRs
rule OwnerHasFullAccessToTheirTreatmentDrugAssets { description: “Allow all participants full access to their assets” participant(p): “org.example.basic.Patient” operation: ALL resource(r): “org.example.basic.TreatmentDrugs” condition: (r.owner.getIdentifier() === p.getIdentifier()) action: ALLOW } rule emergencydoctorHassAccessToPatientLabTest { description: “Allow all participants full access to their assets” participant(g): “org.example.basic.EmergencyDoctor” operation: READ resource(r): “org.example.basic.LabTest” condition: (r.emergencyAcces===true) action: ALLOW } rule emergencydoctorHassAccessToPatientTreatmentDrugs { description: “Allow all participants full access to their assets” participant(g): “org.example.basic.EmergencyDoctor” operation: READ resource(r): “org.example.basic.TreatmentDrugs” condition: (r.emergencyAcces===true) action: ALLOW }

**Table 4:** A comparative analysis of the proposed framework vs the state-of-the-art systems.

Healthcare System Name	Patient Identity	Immutability	Data Auditing	Smart Contracts	Access Control
Our framework	✓	✓	✓	✓	✓
Xiao et al., 2016 [34]	✓	✓	*×*	×	×
Hussein et al., 2018 [38]	✓	✓	*×*	×	×
Dagher et al., 2018 [39]	✓	✓	*×*	✓	×
Chen et al., 2019 [40]	✓	✓	*×*	×	×
Zhang et al., 2018 [41]	✓	✓	✓	✓	×

## Data Availability

Not applicable.
